# The Suture-Clamp Operation for Hemorrhoids

**Published:** 1897-02

**Authors:** Llewellyn Eliot


### THE SUTURE-CLAMP OPERATION FOR HEMORRHOIDS.

BY LLEWELLYN ELIOT, M. D.

It is not my intention, at this time, to discuss the various operations for the cure of hemorrhoids, but simply to call attention to an operation which I have successfully performed several times during the past year. The operation and the suture clamp, so far as I am aware, originated with Dr. William Erwin, of Walter's Park, Pa., and should receive a more extended recognition, as an examination of the instrument will illustrate the simplicity of the procedure.

The clamp consists of two arms bent at a right angle at the lower end. This bent portion, which is about one and one-half inches long, is provided with a shoulder half an inch high and perforated by five small openings. The shafts above the angle are pivoted and serrated to receive a ring which regulates compression and bleeding.

To do this operation, the patient is prepared by having the bowel well flushed; the legs are held with a leg-holder; the sphincter is well dilated, the hemorrhoid seized with forceps and well drawn down, the clamp is then applied as near the base of the hemorrhoid as possible and the ring slipped along the serrations until we have a sufficient amount of compression to control hemorrhage; with a needle, threaded with silk or catgut, the hemorrhoid is then pierced through the needle holes of the clamp When the sutures are placed, the hemorrhoid is cut off with either knife or scissors, the sutures tied and the clamp removed. The stump is dressed either with aristol, acetanilid, iodoform, or other antiseptic material and returned to the bowel should it not return of itself; gauze, absorbent cotton and a T-bandage complete the dressing. Where the hemorrhoid is longer, that is to say, has a broad base, broader than the blade of the clamp is long, the hemorrhoid must be removed in sections, one section being completed to the tying of the last stitch before the second section is attacked; the reason for leaving the last stitch incomplete is that we may have a certain amount of control of the remaining part and then again, the clamp can be placed more satisfactorily. It is surprising to see what a small amount of blood is lost in this procedure, and if the sphincter has been well dilated there is a comparative absence of pain following the operation. Convalescence is usually very rapid, there is but a short detention from business, the sutures are removed on the fifth or sixth day and the wound is then nearly healed. Should the stump remain large, a few applications of a mildly astringent solution of alum or tannic acid will contract them and they finally disappear. Chloroform must be administered to properly perform this, as well as any other operation about the rectum.

No case histories are cited here, but I have removed by this operation seven hemorrhoids at a sitting, when the anus resembled a link of sausage flanked with grapes and cherries. This was more than I would have dared to do under any other operation on account of the troublesome hemorrhage attending the use of the clamp and cautery, the incision, or the Whitehead, or the excessive sloughing which would have attended the injection method. In every case the patient has been discharged before the end of the second week and has remained cured, if the expiration of a year without return may be regarded as a cure. There is no effort made to control the action of the bowels after the third day, when they may be allowed to move. The diet is restricted for the first few days to liquids or semi-fluids; then all restriction is removed. The advantages of this operation are:

1. It is almost bloodless.
2. It does not present a sloughing surface.
3. It heals rapidly.
4. It removes the entire hemorrhoid.
5. It does not affect the tonicity or the calibre of the bowel.—*National Medical Record.*

